# Toxic megacolon: a potentially lethal condition. Case report

**DOI:** 10.1093/jscr/rjae403

**Published:** 2024-06-11

**Authors:** José Jesús Fabián-Méndez, Quitzia Libertad Torres-Salazar

**Affiliations:** Institute of Security and Social Services for State Workers, Tijuana, Baja California, Mexico; Faculty of Medicine and Nutrition, Juárez University of the State of Durango, Durango, Durango 34000, Mexico

**Keywords:** toxic megacolon, ulcerative colitis, atypical presentation

## Abstract

Toxic megacolon denotes an abrupt non-obstructive distension of the colon, accompanied by systemic signs of toxicity. Mortality rates can soar as high as 7.9%. While primarily linked with chronic bowel conditions, the incidence attributed to *Clostridioides difficile* has surged due to the indiscriminate use of broad-spectrum antibiotics. Surgical intervention becomes necessary in the majority of cases. Herein, we illustrate the case of a 50-year-old female presenting with episodic epigastric pain lasting 9 h, vomiting, and watery bowel movements, devoid of peritoneal irritation findings and lacking a history of chronic intestinal inflammation. Under certain circumstances, toxic megacolon may manifest atypically, underscoring the importance of conducting a comprehensive medical history and clinical assessment. Moreover, it is imperative to solicit pertinent paraclinical investigations to address the patient holistically and foster a favorable clinical outcome.

## Introduction

Toxic megacolon is a potentially lethal condition characterized by acute colonic dilatation, exceeding 6 cm in diameter, with a loss of the haustras evident on radiological evaluation in cases of severe colitis. The precise incidence in the general population remains unknown. However, those at greatest risk include individuals with inflammatory bowel disease (IBD), particularly in the early stages of the disease [1]. The incidence associated with *Clostridioides difficile* has increased due to the overuse of broad-spectrum antibiotics [2].

Despite its low frequency, associated outcomes remain disappointing, with an in-hospital mortality rate of 7.9% [3]. The diagnosis of toxic megacolon is established following the clinical criteria outlined by Jalan. These criteria include a body temperature >38.6°C, a heart rate >120 beats per min, a white blood cell count >10 500/L with a left shift, and a 60% decrease in hemoglobin or hematocrit. The presence of at least three of the four criteria mentioned above is required, along with confirmation of colonic distension by imaging studies [4]. Here, we present a clinical case of toxic megacolon with an atypical clinical manifestation. The work has been reported in line with the SCARE criteria [5].

## Clinical case presentation

This report details the case of a 50-year-old female with a 21-year history of systemic arterial hypertension and epilepsy since birth. Hospital admission was prompted by 9 h of generalized abdominal pain, primarily in the epigastric region. The pain, of moderate intensity, began subacutely with fluctuating cramps and was accompanied by severe nausea, recurrent vomiting of gastric contents, and watery bowel movements.

Upon hospital arrival, the patient’s vital signs were within normal parameters. Physical examination revealed deep palpatory pain in the epigastric region (moderate intensity) and in the right hypochondrium (mild intensity). A positive Murphy’s sign was observed, but no signs of peritoneal irritation or acute abdomen were evident. Initial laboratory results indicated an elevated white blood cell count (11.9) with a neutrophil percentage of 66.8%. Other values included hemoglobin (13.1) and hematocrit (36.5). General urine examination revealed pathological abnormalities.

**Figure 1:**
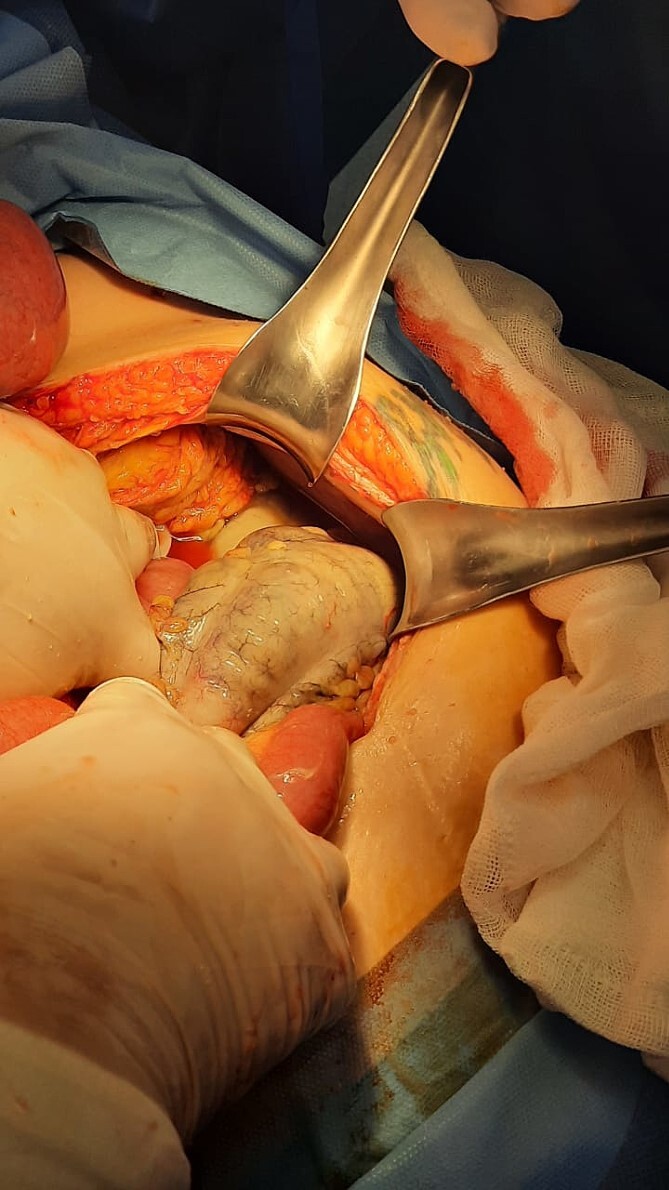
Distended loops, devitalized tissue, and areas of necrosis.

An ultrasound of the liver and biliary tract suggested findings consistent with non-acute chronic cholecystitis. Due to an unfavorable clinical course, a contrasted computed axial tomography scan was performed, revealing generalized distension of the small and large bowel, with hydro-aerial levels predominantly in the colon. Consequently, surgical intervention was pursued, involving an exploratory laparotomy that revealed abundant, purulent, and malodorous peritoneal fluid, as well as a colon with distended loops, devitalized tissue, and areas of necrosis in the ascending, transverse, and descending colon (refer to Fig. 1). A total colectomy with ileostomy and distal closure using Hartmann’s procedure in the rectum was performed. The patient experienced a satisfactory recovery and was discharged 7 days after the procedure.

**Figure 2:**
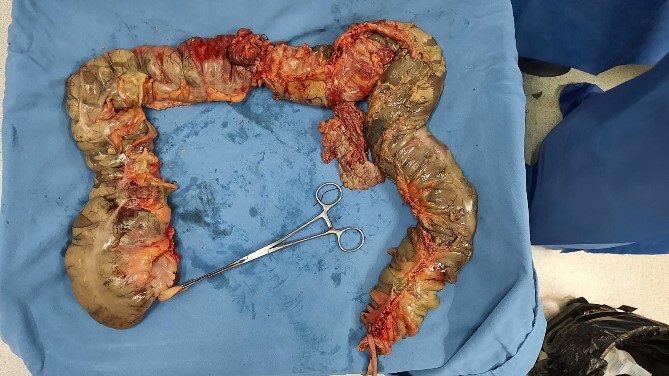
Segment resected and sent to pathology, who reported acute and chronic nonspecific inflammation, necrosis with detachment, and ulceration of the mucosa, as well as diffuse hemorrhage.

**Figure 3:**
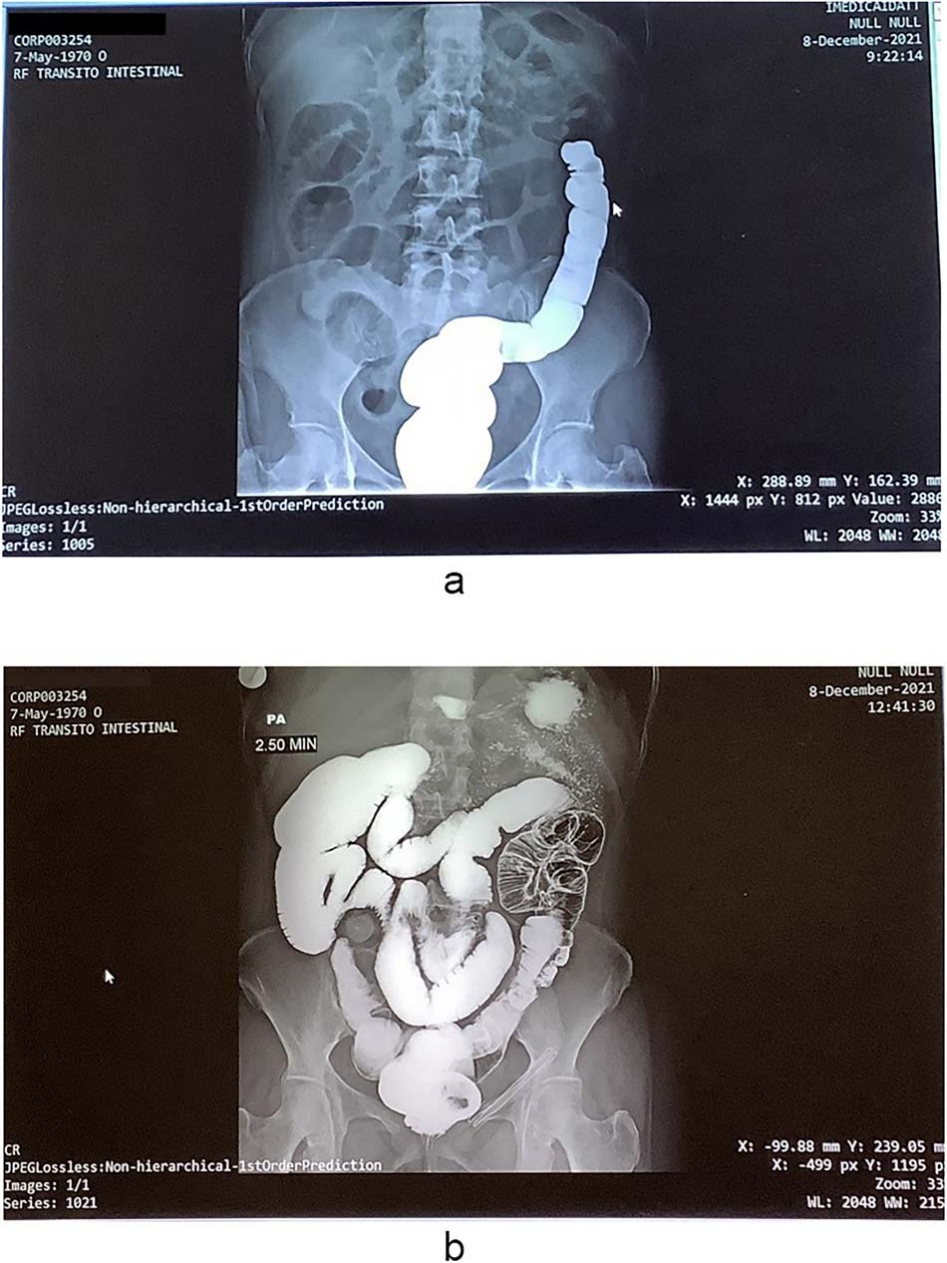
(a) Colonic enema imaging to assess intestinal transit, (b) a generalized expansion of both the small and large intestine is revealed, showing the presence of preponderant hydro-aerial levels in the colon.

Pathological analysis of the surgical specimen revealed acute and chronic nonspecific inflammation, necrosis with detachment, and ulceration of the mucosa, along with diffuse hemorrhage (refer to Fig. 2). Nine months postoperatively, the patient underwent a colon enema for the evaluation of intestinal transit, followed by a subsequent bowel reconnection (Fig. 3). This reconnection involved a latero-lateral anastomosis between the terminal ileum and the rectum, performed utilizing a 60 mm COVIDIEN DTS linear stapler. The procedure was completed without perioperative complications, and the patient was discharged 4 days later.

## Discussion

The overall prevalence of toxic megacolon in the general population remains unknown. The variability in clinical presentation further complicates the estimation of actual case frequency. Indeed, underdiagnosis is plausible due to the diverse manifestations of toxic megacolon, necessitating specific studies for accurate confirmation.

This condition affects individuals of both genders and various ages, with a recognized preponderance among those with IBD, particularly in its early stages [6]. The patient in this instance was unaware of a history of chronic IBD. However, the histopathological report revealed evidence of an acute and chronic nonspecific process, necrosis with detachment and ulceration of the mucosa, as well as diffuse hemorrhage, without prior symptom presentation.

As inflammation progresses, neutrophils invade the muscle layer, causing further damage by releasing proteolytic enzymes, cytokines, and leukotrienes, ultimately leading to dysmotility and colonic dilatation [7]. Several predisposing factors for the development of toxic megacolon have been described, especially in patients with IBD. These factors include changes in the treatment or dosage of background medications for IBD, colonic scans, consumption of medications with the ability to decrease intestinal motility, hypokalemia, intestinal infections, and smoking cessation [8].

Initial therapy involves supportive measures and medical management. Surgical intervention should be anticipated from the outset of medical management. Currently, the preferred surgical option is subtotal colectomy with ileostomy, often complemented by procedures such as Hartmann’s, sigmoidostomy, or rectostomy [4].

## Conclusions

Toxic megacolon, a grave and potentially lethal condition, necessitates meticulous clinical attention. Despite the unknown exact incidence in the general population, the elevated in-hospital mortality rate underscores the imperative for precise diagnosis and prompt therapeutic strategies. The clinical case presented underscores the significance of early surgical intervention, not only to enhance clinical outcomes but also to emphasize the relevance of a multidisciplinary approach in managing this complex medical entity.
